# Effects of the Natural Peptide Crotamine from a South American Rattlesnake on *Candida auris*, an Emergent Multidrug Antifungal Resistant Human Pathogen

**DOI:** 10.3390/biom9060205

**Published:** 2019-05-28

**Authors:** Caroline Dal Mas, Luana Rossato, Thaís Shimizu, Eduardo B. Oliveira, Pedro I. da Silva Junior, Jacques F. Meis, Arnaldo Lopes Colombo, Mirian A. F. Hayashi

**Affiliations:** 1 Departamento de Farmacologia, Escola Paulista de Medicina (EPM), Universidade Federal de São Paulo (UNIFESP), São Paulo SP 04038-032, Brazil; carolinedalmas88@gmail.com (C.D.M.); thaiisyumi@gmail.com (T.S.); 2 Departamento de Medicina, Escola Paulista de Medicina (EPM), Universidade Federal de São Paulo (UNIFESP), São Paulo SP 04038-032, Brazil; luana.farma@hotmail.com; 3 Departamento de Bioquímica e Imunologia, Universidade de São Paulo (USP-RP), Ribeirão Preto SP 14049-900, Brazil; ebdolive@fmrp.usp.br; 4 Special Laboratory for Applied Toxinology (LETA), Center of Toxins, Immune-Response and Cell Signaling (CeTICS), Butantan Institute, São Paulo SP 05503-900, Brazil; pisjr@butantan.gov.br; 5 Center of Expertise in Mycology Radboudumc/CWZ, Department of Medical Microbiology and Infectious Diseases, Canisius-Wilhelmina Hospital (CWZ), 6532 Nijmegen, The Netherlands; j.meis@cwz.nl

**Keywords:** rattlesnake venom toxin, crotamine, antimicrobial peptide, multiresistant strain, fungus, *Candida* spp.

## Abstract

Invasive *Candida* infections are an important growing medical concern and treatment options are limited to a few antifungal drug classes, with limited efficacies depending on the infecting organism. In this scenario, invasive infections caused by multiresistant *Candida auris* are emerging in several places around the world as important healthcare-associated infections. As antimicrobial peptides (AMPs) exert their activities primarily through mechanisms involving membrane disruption, they have a lower chance of inducing drug resistance than general chemical antimicrobials. Interestingly, we previously described the potent candicidal effect of a rattlesnake AMP, crotamine, against standard and treatment-resistant clinical isolates, with no hemolytic activity. We evaluated the antifungal susceptibility of several *Candida* spp. strains cultured from different patients by using the Clinical and Laboratory Standards Institute (CLSI) microdilution assay, and the antifungal activity of native crotamine was evaluated by a microbial growth inhibition microdilution assay. Although all *Candida* isolates evaluated here showed resistance to amphotericin B and fluconazole, crotamine (40–80 µM) exhibited in vitro activity against most isolates tested. We suggest that this native polypeptide from the South American rattlesnake *Crotalus durissus terrificus* has potential as a structural model for the generation of a new class of antimicrobial compounds with the power to fight against multiresistant *Candida* spp.

## 1. Introduction

Infections caused by *Candida* spp. have progressively increased over the last decades, and this phenomenon is mainly associated with the increasing number of critically ill patients exposed to invasive medical procedures, extensive use of broad-spectrum antibiotics, and treatment with immunosuppressants, which notably increase the risk in patients with neoplastic and/or degenerative diseases [1]. All this had contributed to the promotion of *Candida* spp. infections as a public health concern in Europe, the Americas, and Asia [2,3,4]. In this context, *Candida auris* infection has emerged as a serious problem in intensive care units (ICUs) of many countries, due to its reported emerging antifungal resistance, its easy transmission among patients in ICUs, because it is not easily identified by the most widely used phenotypic identification procedures employed in most clinical laboratories, and because it causes severe infection [3,4,5]. Crude mortality rates range from 32% to 66%, depending on the general clinical conditions and age of the patients, geographic region of the incidence, and clinical management of the infection [5,6]. Unfortunately, antifungal therapy against invasive infections caused by this emerging pathogen may be limited. Up to 90% of isolates are resistant to fluconazole (FLC), and 50% have reduced susceptibility to voriconazole, as demonstrated by elevated minimum inhibitory concentrations (MICs) [7]. Currently, echinocandins are recommended for the treatment of *C. auris* infections [3]. However, elevated MICs secondary to hot-spot regions in the FKS genes (*FKS1* and *FKS2*) have been reported in some *C. auris* isolates [7], raising the possibility of pan-drug resistance [4]. Consequently, there is a clear need for identifying new therapeutic candidates and novel treatment strategies to combat infections caused by this emerging pathogen. Identification of natural products with antimicrobial activity against these pan-resistant isolates is one important strategy to be considered. In fact, the value of searching for novel antimicrobial compounds from natural sources has been widely discussed [8].

The native polypeptide crotamine from the venom of the South American rattlesnake *Crotalus durissus terrificus* was originally isolated in the late 1950s [9,10]. However, its real contribution to the envenoming process remains largely unknown, in contrast to the well-exploited molecular pathways underlying its assigned roles in skeletal muscles [11] and as a theranostic agent against cancer [12,13]. The peculiar (three-dimensional) structural similarity of crotamine with the human antimicrobial peptide (AMP) beta defensins [14] stimulated us to study and demonstrate the candicidal effect of crotamine against non-resistant reference strains and against the treatment-resistant clinical isolates, and more importantly, with no hemolytic activity [15].

Defensins are members of a class of multifaceted AMPs that are efficient in killing most microbes, and for which the development of resistance is rare, mainly due to their characteristic mechanism(s) of action based on the interaction of defensins with the lipid bilayer of cell membranes [16,17,18], as also demonstrated by us for crotamine [19]. Interestingly, recent reports have identified human beta defensins as a new type of potassium ion channel inhibitors [20,21], as it was also proposed for crotamine [11,22,23], confirming the striking degree of structural and phylogenetic congruence with functional reciprocity between these antimicrobial polypeptides. The perspective of using host defense AMPs for treating infections caused by bacteria, viruses, or fungi is seriously considered lately by many, mainly due to the emergence of drug-resistance mechanisms, which threaten the efficacy of all current antimicrobial agents [24].

Therefore, in the present work, we evaluated the antimicrobial efficacy of several natural peptides from different sources (including animals and plants), with a special focus on a native AMP from a South American rattlesnake venom, against clinical multidrug-resistant *C. auris* and related emergent species exhibiting phenotypes of multidrug-resistance isolated from different geographic regions.

## 2. Materials and Methods

### 2.1. Crotamine

The native crotamine was extracted and purified from the crude venom of rattlesnakes *C. durissus terrificus* by Dr. Eduardo Oliveira from the Faculdade de Medicina de Ribeirão Preto, São Paulo University-Ribeirão Preto (USP-RP) (authorization: No. 010426/2010 COAPG/DABS/CNPq; term of concession No. 20100104268), essentially as previously described [9]. The other natural peptides evaluated here were isolated and characterized (sequence determination) essentially as previously described [25], and they were all produced by custom chemical synthesis (China Peptides Co., Shanghai, China). Peptide 1 (WRNWE-NH_2_, MW 788.35 Da) and peptide 2 (WRNWE, MW 789.35 Da) were initially identified in the hemolymph of centipedes, *Scolopendra subspinipes* (centipede/Chilopoda), while cheliferin (GAVLDIR, MW 742.4 Da) was from the pseudoscorpion *Chelifer cancroides*. Oligoventin (QPFSLERW, MW 1062 Da) was from the eggs of the Brazilian armed spider *Phoneutria nigriventer* (Ctenidae, Araneomorphae) [25], and comosusin (ITKVFGDEAS, MW 1066.19 Da) was from the peels of pineapple.

### 2.2. Microorganisms

We selected for this study three clinical isolates cultured along the first outbreak of *C. auris* fungemia documented in South America (470/2015, 484/2015, 467/2015), one isolate from the Middle East (CBS 14916), and two *Candida haemulonii* isolates (9873/2014 and 1112/2016) recovered from patients with fungemia admitted at Hospital São Paulo, Brazil [26,27]. In addition, the reference strain *C. auris* CBS 10913 was included in all experiments [28].

### 2.3. Identification of Candida spp. by Sequencing of the ITS Region of rDNA

All isolates were identified at the species level by sequencing of rDNA ITS as previously described [29]. Total genomic DNA was extracted from the *Candida* isolates using PrepMan^®^ Ultra Sample Preparation Reagent (Applied Biosystems Inc., Foster City, CA, USA) according to the manufacturer’s instructions. PCR for the amplification of the ITS region was performed using the forward primer V9G (5′-TTACGTCCCTGCCCTTTGTA-3′) and the reverse primer LS266 (5′-GCATTCCCAAACAACTCGACTC-3′) [30].

### 2.4. Assays for Checking In Vitro Susceptibility of Candida spp. against Antifungal Drugs

The antifungal susceptibility tests were performed using the Clinical and Laboratory Standards Institute (CLSI) microdilution assay [31]. In brief, susceptibility tests were performed in a 96-well plastic microplate containing RPMI 1640 (Sigma-Aldrich Corp., St. Louis, MO, USA) buffered at pH 7.0 with 0.165 M morpholinopropanesulfonic acid (Sigma-Aldrich Corp.), as outlined in the CLSI-M60 document [31]. Plates were incubated at 35 °C for 24 and 48 h. The following antifungal drugs were tested: Fluconazole (FLC) and amphotericin B (AMB) (from Sigma-Aldrich Corp.) and micafungin (MICA, provided by Astellas Pharma Inc., Tokyo, Japan). The final concentrations tested ranged from 0.03 to 16 µg/mL for AMB and MICA and from 0.5 to 64 µg/mL for FLC. The final inoculum density ranged from 0.5 to 2.5 × 10^3^ cells mL^−1^. The MIC results for each agent were determined visually. In the absence of international clinical breakpoint values for AMB and FLC against both species tested, we adapted the reference values for antifungal resistance recently suggested by the Centers for Disease Control and Prevention (CDC) [32] for *C. auris*: FLC ≥ 32 µg/mL and AMB ≥ 2 µg/mL, tested by the Clinical and Laboratory Standards Institute (CLSI) microdilution assay.

### 2.5. Assays for Checking In Vitro Susceptibility of Candida spp. for Natural Peptides Including Crotamine

**Figure 1:**
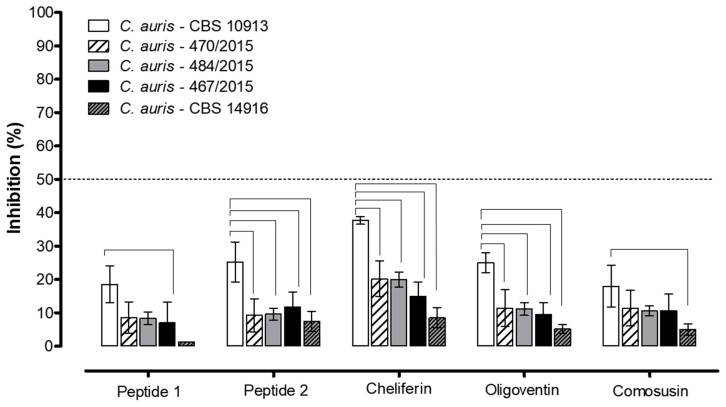
Antifungal activity of several natural peptides against *Candida auris*. *C. auris* (CBS 10913) reference strain (white empty columns), and the clinical multiresistant isolates *C. auris* 470/2015 (white hatched columns) and 484/2015 (light gray columns), *C. auris* 467/2015 (solid black columns), and *C. auris* CBS 14916 (hatched dark gray columns). Peptides 1 and 2 are from the centipede *Scolopendra subspinipes*, cheliferin is from pseudoscorpion *Chelifer cancroides*, oligoventin is from the eggs of the Brazilian armed spider *Phoneutria nigriventer*, and comosusin is from pineapple peels. The percentage of inhibition determined by each peptide (about 1 mM each) was calculated by the ratio between the growth of the microorganisms in the presence/absence (which corresponds to 100% growth) of the peptides. The dashed line indicates approximately 50% inhibition of growth. Differences were considered statistically significant for values of *p* ≤ 0.05 for two-way ANOVA, for the multiple comparisons post-hoc Bonferroni, N = 4, and they are indicated by the bars.

For the antifungal susceptibility assay, the yeast *Candida* spp. was sown with disposable handles in Petri dishes (90 × 15 mm) containing 65 g/L of Sabouraud dextrose agar (Becton, Dickinson and Company, Franklin Lakes, NJ, USA), and the plates were incubated at 37 °C for 48 h. *Candida* spp. isolates were cultured in 5 mL of 25 g/L of PDB medium (Potato Dextrose Broth, Becton, Dickinson and Company), at 37 °C for 18 h in a shaker model New Brunswick™ Innova^®^ 43 (VWR International LLC, Radnor, PA, USA). The in vitro antifungal activity of crotamine and other native peptides was evaluated essentially as described in Yamane et al. [15]. Crotamine was tested in the concentrations of 10, 20, 40, 80, and 160 µM, while other natural peptides were tested in a single concentration (1 mM each), as also described in the Figure 1 legend. These native peptides were added in a 96-well plate containing the isolates of *Candida* spp. (about 2.5–5.0 × 10^3^ cells/100 µL) in PDB medium, before incubation at 37 °C for 24 h. The *Candida* growth rate was determined by measurements at 595 nm using a plate reader SpectraMax (Molecular Devices LLC, San Jose, CA, USA). Briefly, the inhibition of growth was calculated by subtracting the density of the strain in the presence of the peptides studied from the maximum density value of this strain growth in the absence of peptides.

### 2.6. Statistical Analysis

Statistical analysis was performed using the two-way ANOVA test. GraphPad Prism Software (La Jolla, CA, USA) was employed for data analyses. The significance threshold was considered at *p* ≤ 0.05.

## 3. Results

### Susceptibility Tests

Table 1 summarizes the in vitro susceptibilities of all isolates selected for the present study, after evaluation by the CLSI microbroth assay using FLC, AMB, and MICA. The South American and Middle East clinical isolates were resistant to at least two different classes of antifungal drugs. The Asian *C. auris* CBS 10913 reference strain was susceptible to all three antifungal drugs tested here.

The antifungal activity of crotamine and other natural peptides was monitored by a microbial growth inhibition assay in a liquid medium essentially as described earlier [15], as it is the most commonly employed assay to evaluate and express the antifungal activity of native compounds [33], and also to allow direct comparison to previous data [15,25].

The activity of several natural peptides from invertebrate or vertebrate animals and plants were evaluated using fixed concentrations of about 1 mM of each peptide, and they did not show any important or significant inhibition of the growth of resistant *C. auris* clinical isolates from the South America or Middle East outbreaks [26,27], although the Asian *C. auris* CBS 10913 reference was more susceptible to these peptides, with the highest activity observed for cheliferin from the pseudoscorpion *C. cancroides* (Figure 1).

**Figure 2:**
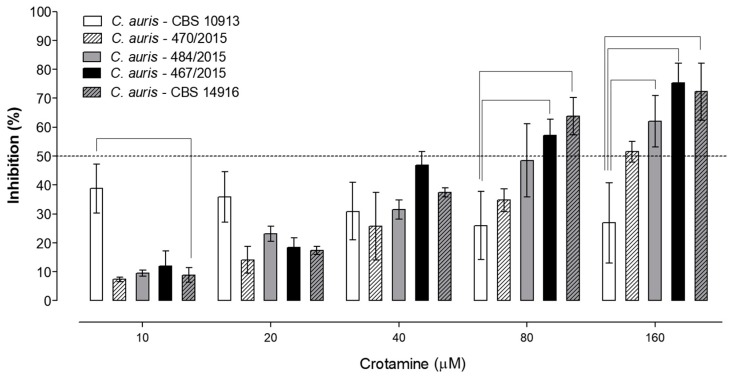
Antifungal activity of native crotamine (10–160 µM) against *C. auris*. *C. auris* (CBS 10913) reference strain (white empty columns), and the clinical multiresistant isolates *C. auris* 470/2015 (white hatched columns) and 484/2015 (light gray columns), *C. auris* 467/2015 (solid black columns), and *C. auris* CBS 14916 (hatched dark gray columns). The percentage of inhibition determined by crotamine was calculated by the ratio between the growth of the microorganisms in the presence/absence (which corresponds to 100% growth) of the peptide. The dashed line indicates approximately 50% inhibition of growth. The bars represent comparison to *C. auris* CBS 10913. Differences were considered statistically significant for values of *p* ≤ 0.05 for two-way ANOVA, for the multiple comparisons post-hoc Bonferroni, N = 6, and they are indicated by the bars.

Although crotamine was also more effective in inhibiting the Asian *C. auris* CBS 10913 reference compared to the resistant *C. auris* clinical isolates from South America or the Middle East outbreaks at low concentration, the activity of native crotamine against these resistant *C. auris* strains was demonstrated with an inhibition of about 50% of the yeast growth for most strains at concentrations of about 80–160 µM (which corresponds to approximately 0.4–0.8 mg/mL of crotamine). At these higher concentrations of crotamine (namely above 80–160 µM), a more pronounced effect of crotamine against the multiresistant *C. auris* 467/2015 and CBS 14916 strains could be observed (Figure 2). Moreover, a trend for a more effective inhibition of resistant *C. auris* 470/2015 and 484/2015 strains compared to the CBS 10913 reference strain could be noticed at 80 µM of crotamine, while at the highest concentration used here (namely 160 µM) the differences were statistically significant for the *C. auris* 484/2015 multiresistant strain also (Figure 2). Although not different among the reference and clinical multiresistant strains, at 40 µM of crotamine close to 40% of inhibition of growth was observed for all evaluated strains, and therefore, we considered as the minimum inhibitory concentration (MIC) values ranging from 40 to 80 µM, which correspond to about 0.2–0.4 mg/mL of crotamine. The in vitro fungicidal activity of crotamine against all these clinical multiresistant *C. auris* strains was demonstrated here.

**Figure 3:**
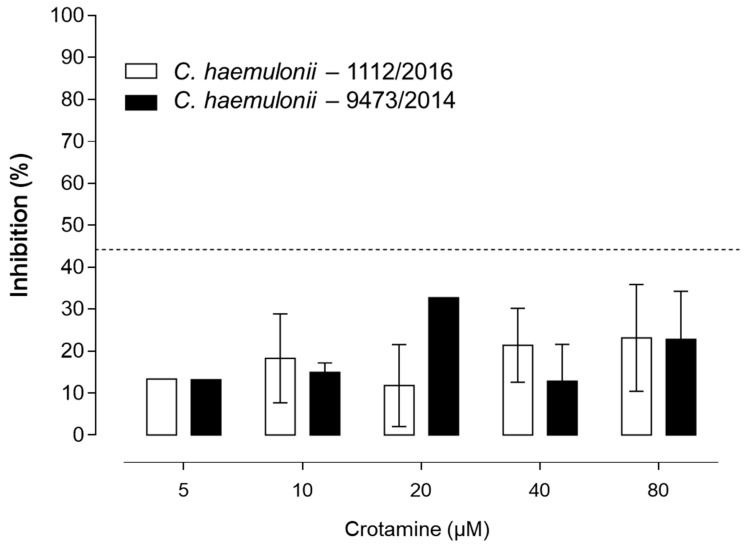
Antifungal activity of native crotamine against *Candida haemulonii*. Different concentrations of native crotamine (from 5 to 80 µM) were tested to evaluate the antifungal activity against the *C. haemulonii* resistant clinical isolates, namely *C. haemulonii* 1112/2016 (white empty columns) and *C. haemulonii* 9873/2014 (solid black columns). Differences were considered statistically significant for values of * *p* ≤ 0.05 for two-way ANOVA (N = 3).

In addition, the closely related resistant *C. haemulonii* clinical isolates (9873/2014 and 1112/2016) were also evaluated with crotamine, but inhibition by less than 40% for concentrations up to 80 µM of crotamine was observed. In addition, no significant differences in inhibition efficiency with increasing concentrations of crotamine could be observed (Figure 3).

## 4. Discussion

*C. auris* is an emergent multiresistant *Candida* species able to disseminate in the hospital environment [3]. Antifungal susceptibility data published so far point out that some *C. auris* strains exhibit elevated minimum inhibitory concentration (MIC) for the three major classes of antifungal drugs, i.e., azoles, polyenes, and echinocandins [7]. *C. haemulonii* complex isolates are considered to be emergent species related to *C. auris* that may also exhibit a multiresistant phenotype to antifungal drugs [2]. So far, several authors have tried to explore different strategies of drug combinations to find new tools to combat this new emerging multiresistant pathogen [34,35,36].

The value of screening natural compounds for searching for new compounds with antimicrobial activity against multiresistant strains is well recognized. However, the several different peptides from invertebrate and plants, with already demonstrated antimicrobial activity, showed important activity only against the Asian *C. auris* CBS 10913 reference strain, with no significant effect against the multiresistant *C. auris* clinical isolates from South America or the Middle East. On the other hand, the effective antifungal activity of crotamine against these multiresistant clinical *C. auris* strains from different patients with fungemia points out the potential of crotamine as a structural model for the development of a new generation of antimicrobial drugs against multidrug-resistant clinical strains. At this point, it is also worth considering that although few clinical strains were evaluated, all those strains were from independent emergences of different clonal populations on different continents [26,27,28].

In the present study, we also decided to evaluate the antifungal activity of crotamine against clinical multiresistant *C. haemulonii*, which is often misidentified by commercial identification methods and presents a multidrug-resistant profile as confirmed here (Table 1) and as described by others [2], but with no important effect in concentrations up to 80 µM of crotamine.

Crotamine is a well-characterized polypeptide with multiple biological activities [37], including the in vitro activity against a selected panel of *Candida* species [23]. Crotamine is also a recognized member of the antimicrobial peptide (AMPs) class whose members are efficient in killing most microbes, and for which the development of resistance is rare, mainly due to the characteristic mechanism(s) of action based on the rapid interaction with and disruption of lipid cell membranes [15,19,38]. AMPs represent therefore a powerful drug candidate with reduced risk of resistance development and potential reduction in the duration of treatment [38].

The action of crotamine on mitochondria was also previously demonstrated by us [13], and phenolic compounds such as flavonoids, when combined with FLC, were demonstrated to show activity against *Candida tropicalis* strains resistant to FLC, by promoting mitochondrial depolarization, apoptosis, and exposure of phosphatidylserine in the plasma membrane [39]. Interestingly, crotamine is also able to promote mitochondrial depolarization and apoptosis [12,13], besides having an affinity for negatively charged lipids such as phosphatidylserine [19,38]. Furthermore, although the lipid-membrane-disrupting activity of crotamine and its shorter derived peptides was previously demonstrated by us [19,38], at this point we cannot simply come to the conclusion that the negative charges in the cell membrane of FLC-resistant strains could be playing a role in the candicidal effect against the *C. auris* resistant strains. In addition, the low ability of native crotamine in inhibiting the closely related *C. haemulonii* resistant clinical isolates also deserves special attention, and further studies are planned to clarify this selective activity of native crotamine only against the *C. auris* clinical strains.

## 5. Conclusions

Based on our present experiments, we suggest that this native polypeptide from the South American rattlesnake has potential as a structural model compound for the generation of a new class of antimicrobial compound with power against multiresistant nosocomial *Candida* strains, representing a possible new road to overcome the microbial resistance challenge against emerging opportunistic human fungal pathogens. At this point, it remains unclear if the concentrations of crotamine required to inhibit *C. auris* and *C. haemulonii* strains could be safely administrated for treating human infections. However, we may certainly suggest that these data encourage further studies to explore the possible use of crotamine as topical antifungal agents or to study the structural aspects of crotamine as a molecular template for modeling and designing of new molecules with higher efficacy against multiresistant clinical strains.

However, further studies are still necessary to determine the molecular mechanism of action underlying the crotamine activity against the multidrug-resistant strains of *C. auris* and its relative inefficiency against the *C. haemulonii* clinical strains, and this may certainly be the next target of our future studies.

## Figures and Tables

**Table 1:** Minimum inhibitory concentrations (MICs) (µg/mL) of fluconazole (FLC), amphotericin B (AMB), and micafungin (MICA) for *Candida* spp. isolates.

Strains	AMB	FLC	MIC
*C. auris* CBS 10913	0.5/0.5 (S)	2/2 (S)	0.03/0.06 (S)
*C. auris* 470/2015	2/2 (R)	>64/>64 (R)	0.06/0.12 (S)
*C. auris* 484/2015	4/4 (R)	>64/>64 (R)	0.06/0.12 (S)
*C. auris* 467/2015	2/4 (R)	>64/>64 (R)	0.06/0.12 (S)
*C. auris* CBS 14916	2/2 (R)	>64/>64 (R)	0.12/0.12 (S)
*C. haemulonii* 9873/2014	1/>16 (R)	8/16 (R)	0.03/0.06 (S)
*C. haemulonii* 1112/2016	1/>16 (R)	8/16 (R)	0.03/0.06 (S)

(R) resistant; (S) sensitive; MIC values are presented as µg/mL determined for 24/48 h incubations.
